# Coronary CT angiography features of ruptured and high-risk atherosclerotic plaques: Correlation with intra-vascular ultrasound

**DOI:** 10.1016/j.jcct.2017.09.001

**Published:** 2017-11

**Authors:** Daniel R. Obaid, Patrick A. Calvert, Adam Brown, Deepa Gopalan, Nick E.J. West, James H.F. Rudd, Martin R. Bennett

**Affiliations:** a Division of Cardiovascular Medicine, University of Cambridge, Addenbrooke's Hospital, Cambridge, UK; b Department of Radiology, Papworth Hospital NHS Trust, Cambridge, UK; c Department of Interventional Cardiology, Papworth Hospital NHS Trust, Cambridge, UK

**Keywords:** Atherosclerosis, Coronary computed tomography angiography, Intravascular ultrasound, Vulnerable plaque, Ruptured plaque

## Abstract

**Background:**

Features of ruptured and high-risk plaque have been described on coronary computed tomography angiography (coronary CTA), but not systematically assessed against intravascular ultrasound (IVUS). We examined the ability of coronary CTA to identify IVUS defined ruptured plaque and Virtual Histology Intravascular Ultrasound (VH-IVUS) defined thin-cap fibroatheroma (TCFA).

**Methods:**

Sixty-three patients (32 with acute coronary syndrome and 31 with stable angina) underwent coronary CTA, IVUS and VH-IVUS. Plaque rupture on CTA was defined as intra-plaque contrast and its frequency compared with IVUS-defined plaque rupture. We then examined the relationship of conventional coronary CTA high-risk features (low attenuation plaque, positive remodeling, spotty calcification and the Napkin-Ring sign) in VH-IVUS-defined TCFA. We compared these with a novel index based on quantifying the ratio of necrotic core to fibrous plaque using x-ray attenuation cut-offs derived from the relationship of plaque to luminal contrast attenuation.

**Results:**

Of the 71 plaques interrogated with IVUS, 39 were ruptured. Coronary CTA correctly detected 13-ruptured plaques with 3 false positives giving high specificity (91%) but low sensitivity (33%). None of the conventional coronary CTA high-risk features were significantly more frequent in the higher-risk (VH-IVUS defined thin-cap) compared with thick-cap fibroatheroma. However, the new index (necrotic core/fibrous plaque ratio) was higher in thin-cap (mean 0.90) vs. thick-cap fibroatheroma (mean 0.59), p < 0.05.

**Conclusions:**

Compared with intravascular ultrasound, coronary CTA identifies ruptured plaque with good specificity but poor sensitivity. We have identified a novel high-risk feature on coronary CTA (necrotic core/fibrous plaque ratio that is associated with VH-IVUS defined-TCFA.

## 1 Introduction

### 1.1 Ruptured plaque

Post–mortem studies reveal that most myocardial infarctions are caused by coronary plaque rupture followed by thrombotic arterial occlusion.^1^ Features of ruptured plaque have been described on coronary computed tomography angiography (coronary CTA) including ulceration and intra-plaque dye penetration but to date have not been tested systematically against the gold standard of intra-coronary imaging.^2^, ^3^ We assessed the accuracy of coronary CTA to detect ruptured plaque identified using intravascular ultrasound in patients with stable angina and acute coronary syndrome (ACS).

### 1.2 Vulnerable plaque

Identification of vulnerable plaques prior to rupture is also of considerable importance. High-risk plaques have characteristic appearances at post-mortem; these thin-cap fibroatheromas (TCFAs) have more necrotic core (NC) than stable lesions, with an overlying thin fibrous cap.^4^ Virtual-Histology intravascular ultrasound (VH-IVUS) is an invasive imaging modality that can identify this vulnerable coronary plaque type.^5^, ^6^ However, a non-invasive alternative is desirable for generalised use. Due to limitations in spatial resolution coronary CTA cannot identify TCFA by direct visualisation of the thin-cap^6^ Some coronary CTA features have been associated with acute coronary syndromes (ACS) including spotty calcification, low attenuation plaque (LAP), positive remodeling index (RI) and the “Napkin-Ring” sign^7^, ^8^ but their ability to act as surrogate markers of TCFA is unknown.

We sought to determine the utility of these previously described high-risk coronary CTA features in identifying TCFA determined by VH-IVUS. Quantifying plaque volume can also improve coronary CTA prediction of ACS^9^ and determining plaque constituent volumes may improve identification of TCFA. However, overlap in the attenuation of fibrous plaque and necrotic core has traditionally limited this.^10^ We previously identified coronary CTA lumen contrast/plaque attenuation ratios that can discriminate fibrous tissue, necrotic core and calcification with minimal overlap.^6^ We investigated whether using these ratios to calculate a novel, non-invasively determined index of necrotic core/fibrous plaque improved coronary CTA identification of TCFA.

## 2 Methods

### 2.1 Patients

Following Ethics Committee approval and informed consent, 63 patients were enrolled scheduled to undergo percutaneous coronary intervention (PCI) for stable angina (n = 31) or ACS (n = 32). Patients with ACS were enrolled within 72 h of symptom onset and all patients underwent coronary CTA immediately before PCI, and IVUS and VH-IVUS during PCI prior to stent implantation. Patients with atrial fibrillation or renal impairment were excluded. IVUS was not possible in 6/63 patients for technical reasons.

### 2.2 Invasive quantitative coronary angiography (QCA)

All plaques were examined by catheter angiography for qualitative markers of rupture (ulceration, intimal flaps, lumen irregularities and visible thrombus)^11^ and were assessed by observers blinded to patient presentation. Culprit plaques were identified using invasive coronary angiography and localising ECG features in ACS patients and stress or ischaemia testing in stable patients. QCA from two orthogonal planes was performed on all culprit plaques (Cardiac Viewer CV-1000, version 2.1.0) to determine lesion length, diameter and stenosis.

### 2.3 Computed tomography angiography

**Fig. 1:**
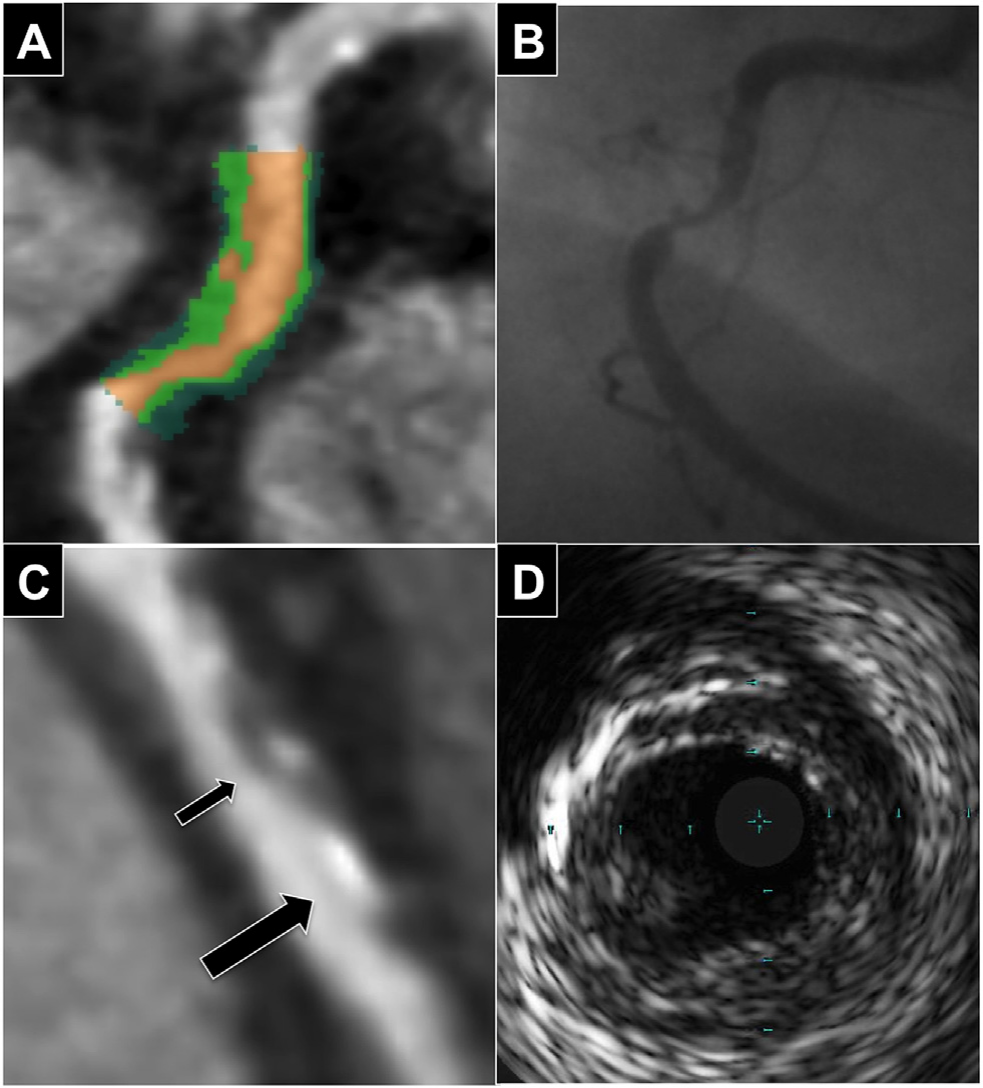
**CTCA, angiographic and IVUS features of plaque rupture. (A)** CTCA image of intra-plaque contrast with colour mapping. **(B)** Corresponding ulceration on coronary angiography. **(C)** Demonstration that intra-plaque contrast (small arrow) can be difficult to distinguish from small calcified plaque (big arrow) on CTCA. **(D)** Corresponding co-registered plaque on IVUS.

**Fig. 2:**
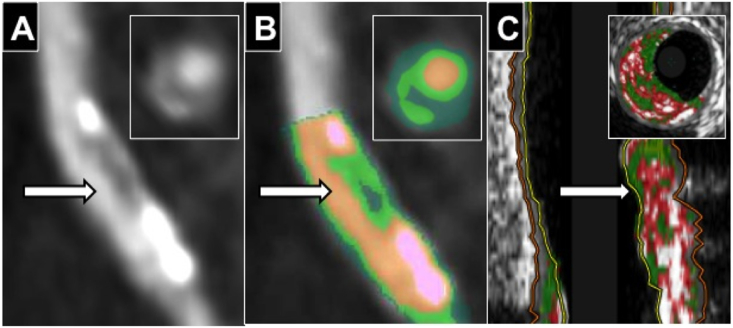
**Coronary CTA Napkin-Ring sign and corresponding plaque map. (A)** ‘Napkin-Ring’ sign with low attenuation plaque surrounding by higher attenuation non-calcified plaque. **(B)** Plaque Map with plaque quantification necrotic core (35.3%-dark green) surrounded by fibrous plaque (51.5%-light green)(orange = lumen, calcified plaque = pink (13.2%) Necrotic core/fibrous plaque ratio = 0.69. **(C)** Co-registered VH-IVUS fibroatheroma (red = necrotic core, green = fibrous plaque and white = calcified plaque). Insets show cross-sectional images at arrows.

Patients underwent retrospectively-gated coronary CTA with ECG-dependent tube current modulation using a Somatom Definition 64-slice dual source CT (Siemens, Germany), pitch 0.20–0.48, collimation 32 × 2  mm × 0.6  mm, tube voltage 120 kV and current 360 mA. Intravenous contrast was injected in a triphasic protocol following a 20 ml timing bolus to assess circulation time. Coronary CTA analysis was performed by a reviewer blinded to the mode of patient presentation. Ruptured plaques were classified on coronary CTA using the previously described features of ulceration (contrast extending beyond the lumen but contiguous with it) or intra-plaque dye penetration (contrast pool within plaque but not contiguous with lumen)(Fig. 1).^3^ High-risk features were defined as: spotty calcification (calcific lesions <3 mm in diameter), positive remodeling (ratio of vessel diameter at lesion site to reference vessel >1.05), low attenuation plaque (LAP)(<30HU), Napkin-Ring sign (low attenuation surrounded by higher attenuation rim <130HU)(Fig. 2).^12^, ^13^ Plaque quantification was performed using semi-automated Circulation III software (Siemens, Germany) with manual correction of vessel contours if required. Volumes of total plaque, non-calcified plaque and % stenosis (100-(lumen volume/vessel volume) x 100) were calculated. Plaque was separated into constituent parts with the software colour-coding each plaque voxel depending on its attenuation to create a Plaque Map allowing visualisation of the plaque components (Fig. 2). The attenuation (HU) cut-offs for each component were calculated according to ratios of luminal contrast and plaque attenuation (necrotic core <0.197, fibrous plaque 0.197–0.470, calcified plaque >1.295) derived using histological validation and described in-detail previously.^6^ For example, an average coronary luminal contrast intensity of 200HU would give an upper limit for necrotic core as 0.197 × 200 = 39HU, whilst for a contrast intensity of 400HU it would be 0.197 × 500 = 79HU. This allows the volumes of necrotic core, fibrous plaque and calcified plaque to be calculated as well as the proposed vulnerability index of necrotic core/fibrous plaque ratio.

### 2.4 Intravascular ultrasound analysis

Intravascular ultrasound (IVUS) and Virtual Histology IVUS (VH-IVUS) data were acquired with Eagle-Eye Gold catheters (Volcano, USA) using a motorized pull-back at 0.5 mm/s. Plaques were examined by IVUS for evidence of rupture, including ulcerated plaque and luminal tears contiguous with an echolucent cavity. Plaques were also classified using VH-IVUS definitions described previously.^14^ Plaques with >10% confluent necrotic core in 3 consecutive frames were classified as fibroatheromas, and separated into thick-cap fibroatheroma (fibrous plaque separating necrotic core from lumen present) and thin-cap fibroatheroma (if there was not).

### 2.5 Co-registration

**Fig. 3:**
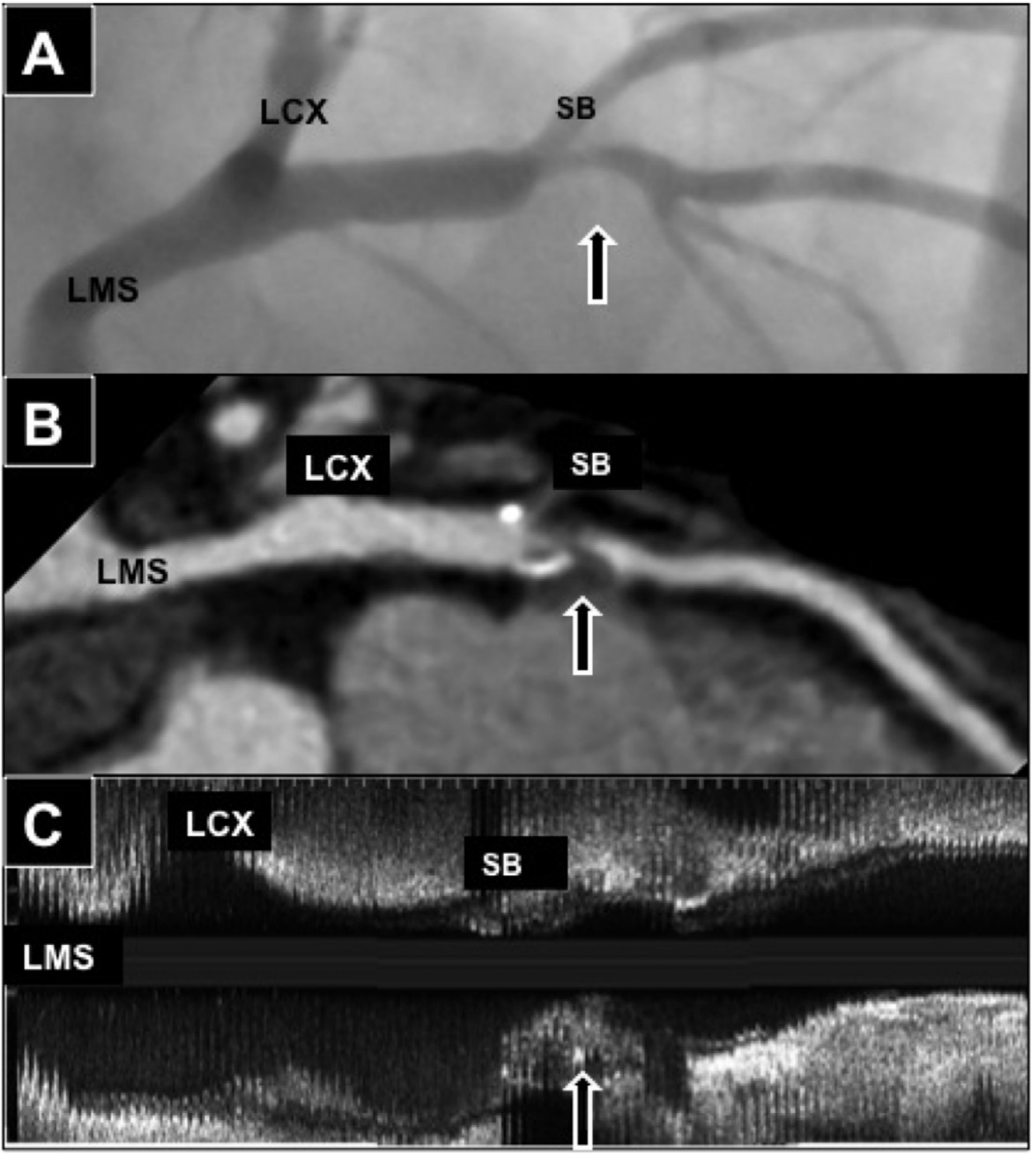
**Co-registration of CT, IVUS and angiography.** Fibroatheroma plaque with large necrotic core in Left anterior descending coronary (Arrow) in **(A)** Catheter angiography **(B)** CT curved multi-planar reformat and **(C)** IVUS longitudinal view. Co-registration performed using distances from Left main stem (LMS), LCX-left circumflex artery and SB-side branch.

Curved multi-planar coronary artery reconstructions were compared with longitudinal reconstructed IVUS datasets using at least 2 orthogonal catheter angiography views. Plaque locations were co-registered by triangulation using reference measurements from coronary ostia and side branches (Fig. 3).

### 2.6 Statistical analysis

Statistical analysis was performed using GraphPad Prism version 5.00 (GraphPad Software, San Diego, CA). Continuous variables are presented as mean±SDs and compared using unpaired *t*-test with p < 0.05 considered statistically significant. Diagnostic accuracy was expressed as area under receiver operating characteristic curves (ROC). Categorical variables are presented as percentages with accuracy expressed as point estimates of sensitivity, specificity and diagnostic accuracy.

## 3 Results

### 3.1 Coronary CTA features of IVUS-defined ruptured plaque

The mean heart rate of the patients was 61.1 beats per minute ±10.8. All 63 coronary CTA scans were of sufficient quality for plaque analysis. IVUS was performed in 57 patients yielding 71 plaques co-registered with coronary CTA for interrogation. 25 from the proximal Left Anterior Descending (LAD) coronary artery, 13 from the Mid LAD, 2 from the first diagonal branch, 9 from the Proximal Left Circumflex (LCX), 2 from the Mid LCX, 2 from the First Obtuse Marginal, 7 from the Proximal Right Coronary Artery (RCA), 7 from the Mid RCA, and 3 from the Distal RCA. Of these plaques, 39 had evidence of rupture and 32 were intact by IVUS. Coronary CTA features of rupture (ulceration or intra-plaque dye penetration) allowed correct identification of 13 of the ruptured plaques (33%) but led to false positive identification in 3 plaques (9%). This gives coronary CTA a sensitivity to identify ruptured plaque of 33% and a specificity of 91%. This compares with invasive angiography, which identified 19 (49%) of the ruptured plaques with 4 (13%) false positives (sensitivity 49%, specificity 88%). Of the high-risk features coronary CTA features, the Napkin-Ring sign was more common in ruptured than intact plaque (56% vs. 31%, p = 0.03) but spotty calcification (54% vs. 47%, p = 0.57), low attenuation plaque (56% vs. 77%, p = 0.07) and remodeling index (44% vs. 59%, p = 0.21) were all not significantly different.

### 3.2 Coronary CTA features of VH-IVUS-defined vulnerable plaque

#### 3.2.1 VH-IVUS defined necrotic core

VH-IVUS analysis was available in 69 of the plaques. Fifty-five contained a confluent necrotic core >10% plaque volume on VH-IVUS, classifying them as fibroatheromas. The remaining 14 plaques did not contain a confluent necrotic core (6 were fibrocalcific plaques and 8 were pathological intimal thickening). Examining the ability of coronary CTA to distinguish plaques with VH-IVUS defined necrotic core, we found when compared with plaques without confluent necrotic cores, coronary CTA-defined lesion length, stenosis, total or non-calcified plaque volume, spotty calcification and increased RI were similar, however Plaques with VH-IVUS detected necrotic cores (fibroatheromas) more often contained coronary CTA defined Napkin-Ring sign (56% vs. 0%, p < 0.01), and LAP (76% vs. 36% p = 0.01)(Table 1). This meant using the coronary CTA criteria LAP detected VH-IVUS-defined necrotic core (fibroatheromas) with a sensitivity of 76% and specificity of 64%. The Napkin-Ring sign reduced sensitivity to 56%, but increased specificity to 100%.

**Table 1:** **Geometrical features and plaque composition of VH-IVUS-defined plaque types.** (V ref–reference vessel diameter, V lesion–vessel diameter at lesion, RI–remodeling index, Spotty Ca–spotty calcification, LAP–low attenuation plaque, Ca–calcified plaque volume, Fib–fibrous plaque volume and NC–necrotic core volume).

	VH-IVUS-defined plaque type					
	Necrotic core absent	Necrotic core present	P Value	Fibroatheroma		P Value
				Thick-Cap	Thin-Cap	
**Conventional coronary CTA**						
Length (mm)	19.6 ± 12.4	18.3 ± 6.7	0.70	21.7 ± 8.3	17.3 ± 6.0	0.11
Spotty Ca %	43	53	0.53	67	49	0.28
LAP %	36	76	**0.01**	75	77	0.91
+ve RI	36%	60%	0.10	58%	58%	0.99
Napkin-Ring %	0	56	**<0.01**	33	63	0.08
**Plaque Quantification**						
Lumen (mm^3^)	167.1 ± 139.2	150.6 ± 81.7	0.68	180.7 ± 94.0	142.0 ± 76.9	0.21
Vessel (mm^3^)	396.4 ± 322.4	421.6 ± 197.0	0.78	458.8 ± 250.4	411.5 ± 181.6	0.56
Stenosis %	59.1 ± 13.9	63.3 ± 15.8	0.33	57.9 ± 19.7	64.8 ± 14.4	0.27
Plaque (mm^3^)	192.0 ± 160.1	215.3 ± 106.1	0.61	236.7 ± 134.7	209.3 ± 97.8	0.52
Non-Ca Pl (mm^3^)	161.3 ± 121.1	200.8 ± 105.1	0.29	211.3 ± 128.1	197.8 ± 99.3	0.74
**Plaque Map Features**						
NC (mm^3)^	62.8 ± 61.1	90.5 ± 58.5	0.14	77.2 ± 52.1	94.2 ± 60.2	0.35
NC%	31.4% ± 10.8	40.2% ± 12.1	**0.01**	32.4% ± 9.9	42.4 ± 11.9	**<0.01**
Ca (mm^3)^	30.6 ± 46.3	14.5 ± 21.9	0.22	25.4 ± 35.2	11.4 ± 15.9	0.21
Ca%	11.3% ± 13.7	7.6% ± 11.4	0.37	10.5% ± 11.6	6.8% ± 11.4	0.34
Fib (mm^3)^	98.6 ± 66.4	111.0 ± 59.2	0.53	137.2 ± 85.8	103.6 ± 48.2	0.22
Fib%	57.3% ± 12.4	52.5% ± 11.7	0.20	58.7% ± 11.4	50.8% ± 11.3	**0.048**
NC/Fib	0.58 ± 0.27	0.83 ± 0.40	**<0.01**	0.59 ± 0.27	0.90 ± 0.40	**<0.01**

P values that are statistically significant (<0.05) are in bold.

#### 3.2.2 VH-IVUS defined thin-cap fibroatheroma

VH-IVUS-defined fibroatheromas were further divided into thin-cap (n = 43) or thick-cap (n = 12) fibroatheromas. The previously described coronary CTA high-risk features were not significantly more common in the vulnerable thin-cap compared with thick-cap fibroatheromas, including positive RI (58% vs. 58%, p = 0.99), LAP (77% vs. 75%, p = 0.91), spotty calcification (49% vs. 67%, p = 0.28), Napkin-Ring sign (63% vs. 33%, p = 0.08) and non-calcified plaque volume (197.8 ± 99.3 mm^3^vs. 211.3 ± 128.1 mm^3^, p = 0.74) (Table 1). In contrast, using coronary CTA Plaque Maps to define plaque constituent volumes VH-IVUS defined TCFAs had lower fibrous plaque % (50.8% ± 11.3 vs. 58.7% ± 11.4, p = 0.048), higher necrotic core % (42.4 ± 11.9 vs. 32.4% ± 9.9, p < 0.01), and higher NC/Fib ratio (0.90 ± 0.40 vs. 0.59 ± 0.27, p < 0.01) compared with thick-cap fibroatheromas (Table 1).

**Fig. 4:**
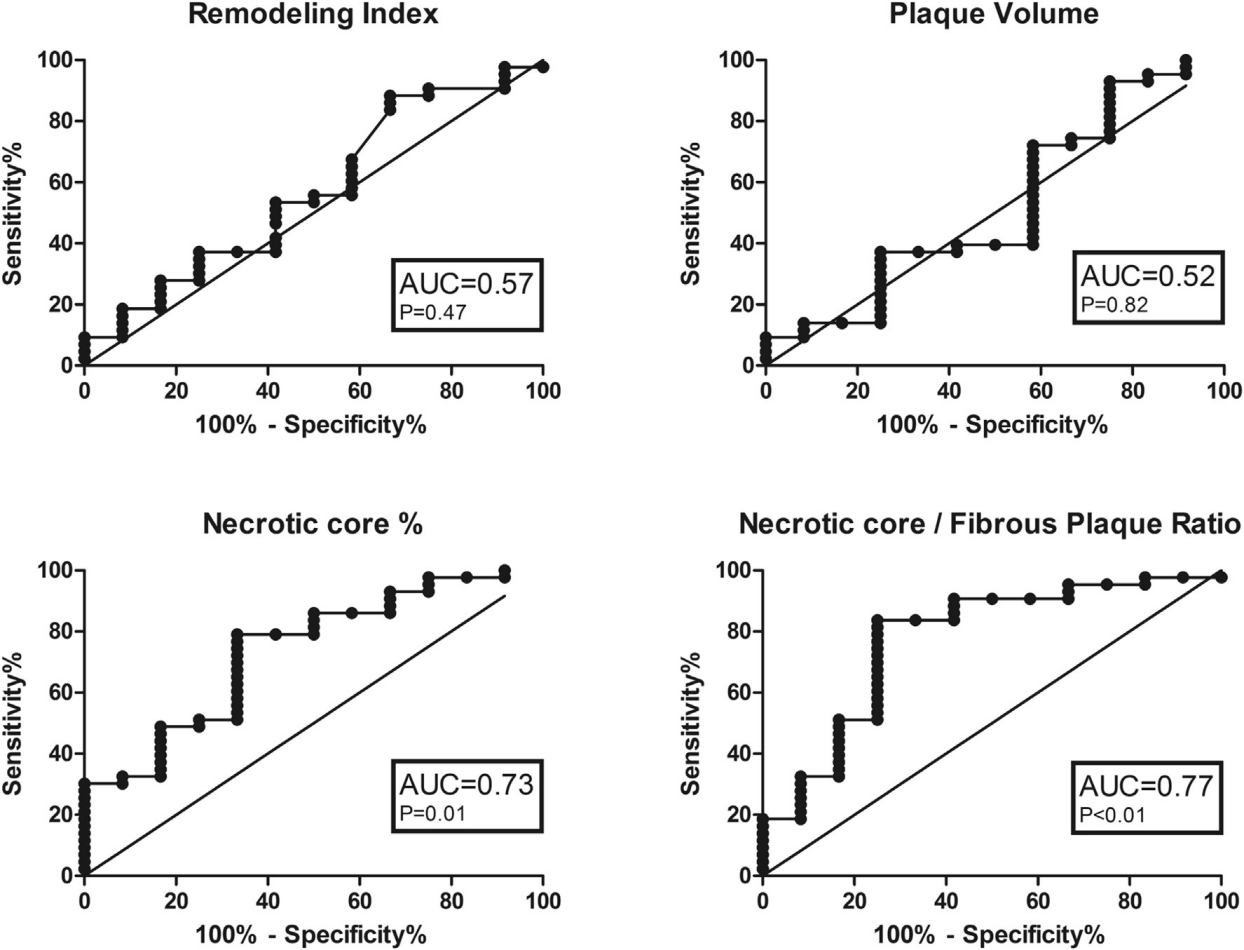
**Receiver operator characteristics for plaque characteristics that differentiate thick and thin-capped fibroatheroma.** AUC-area under curve.

ROC curves were created to determine whether any characteristics had utility in identifying VH-IVUS defined vulnerable plaque. The area under the curve (AUC) values for conventional coronary CTA revealed that none were statistically significant in discriminating between thin-cap vs. thick-cap fibroatheromas, including remodeling index (0.57, p = 0.42), total plaque volume (0.52, p = 0.82) and non-calcified plaque volume (0.50, p = 0.97). However, the AUC values for Plaque Map-derived NC% (0.73, p = 0.01) and NC/Fib ratio (0.77, p < 0.01) were significant (Fig. 4).

#### 3.2.3 Culprit plaque analysis in ACS and stable angina patients

Finally, we analysed the culprit lesions of 63 patients (31 stable angina, 32 ACS (non ST-elevation MI)). Conventional coronary risk factors, angiographically derived lesion length and diameter stenosis, and coronary CTA Plaque Map derived vessel size, percentage stenosis, and total plaque volumes were all similar in ACS vs. stable angina culprit lesions (Table 2). Angiographic features of rupture (Fig. 2) were detected in 66% of ACS culprit plaques compared with 10% stable angina culprit plaques, p < 0.01) whilst coronary CTA features of rupture were found in the culprit plaque of 44% of ACS vs. 6% in stable angina, p < 0.01 (Table 2). Conventional coronary CTA high-risk features were more common in ACS compared with stable angina culprit plaque including; Spotty calcification (63% vs. 35%, p = 0.03), LAP (88% vs. 58%, p < 0.01), positive RI (80% vs. 22%, p < 0.01) and the Napkin-Ring sign (56% vs. 23%, p < 0.01). Coronary CTA Plaque Map derived non-calcified plaque volumes were higher in ACS culprit plaque (236.9 mm^3^ ± 112.9 vs. 163.9 mm^3^ ± 97.8, p < 0.01)(Table 2). ACS culprit plaques also had higher percentage of necrotic core (44.2% ± 12.7 vs. 34.4% ± 13.2, p < 0.01), but lower calcified plaque percentages (4.0% ± 6.1 vs. 15.0% ± 19.4, p < 0.01)(Table 2). Fibrous tissue percentages were similar, however, NC/Fib ratio was significantly higher in ACS culprit plaques (0.94 ± 042 vs. 0.73 ± 0.31, p = 0.03).

**Table 2:** **Demographic details, invasive angiography and coronary CTA analysis of culprit plaques from patients presenting with ACS or stable angina.** Vol–volume, RI–remodeling index, Spotty Ca–spotty calcification, LAP–low attenuation plaque, Non-Ca pl–non-calcified plaque, Ca–calcified plaque, Fib–fibrous plaque and NC–necrotic core.

Demographics	Stable (n = 31)	ACS (n = 32)	P value
Age	63.2 ± 10.8	62.5 ± 10.6	0.83
Male %	76	75	0.95
Cholesterol (mmol/L)	4.2 ± 1.7	4.6 ± 1.1	0.40
Diabetes %	14	6	0.45
Hypertension %	68	47	0.18
Previous MI %	4	6	0.71
Current smoker %	11	33	0.08

**Invasive Catheter Angiography**			
Lesion length (mm)	16.3 ± 8.2	18.7 ± 9.3	0.28
Diameter stenosis %	65 ± 17	73 ± 22	0.14
Rupture %	10	66	**<0.01**

**Coronary CTA Features**			

Spotty Ca %	35	63	**0.03**
LAP %	58	88	**<0.01**
+ve RI	22%	80%	**<0.01**
Napkin-Ring %	23	56	**<0.01**

**Plaque Quantification**			
Lumen vol (mm^3^)	141.5 ± 97.5	141.9 ± 88.5	0.99
Vessel vol (mm^3^)	391.8 ± 247.3	462.2 ± 204.6	0.22
Stenosis %	64.1 ± 16.6	69.3 ± 17.3	0.23
Plaque vol (mm^3^)	202.3 ± 131.7	248.4 ± 114.2	0.14
Non-Ca pl vol (mm^3^)	163.9 ± 97.8	236.9 ± 112.9	0.08

**Plaque Map Features**			
NC vol (mm^3^)	71.0 ± 52.1	112.6 ± 65.2	**<0.01**
NC%	34.4 ± 13.2	44.2 ± 12.7	**<0.01**
Ca vol (mm^3^)	36.4 ± 67.8	11.5 ± 17.6	**0.03**
Ca%	15.0 ± 19.4	4.0 ± 6.1	**<0.01**
Fib vol (mm^3^)	92.9 ± 50.3	125.0 ± 64.0	**0.03**
Fib%	41.2 ± 11.3	42.3 ± 11.4	0.70
NC/Fib	0.73 ± 0.31	0.94 ± 042	**0.03**
CT detected Rupture %	6%	44%	**<0.01**

P values that are statistically significant (<0.05) are in bold.

## 4 Discussion

Previous studies have identified ulceration and intra-plaque dye penetration as coronary CTA markers of disrupted plaques with a sensitivity of 61–81% and specificity of 79–95%.^3^, ^15^ However, these studies compared coronary CTA with invasive coronary angiography, which is not the gold standard for the diagnosis of disrupted or ruptured plaques. We have shown that when compared with the gold standard of intravascular imaging (intravascular ultrasound) invasive coronary angiography only had a sensitivity of 49% and a specificity of 81% to detect ruptured plaque. Ours is the first study to systematically compare coronary CTA features of disrupted plaque with intravascular ultrasound. We found intra-plaque contrast on coronary CTA had a high specificity of 91% but a low sensitivity of 33% to detect ruptured plaque. The Napkin-Ring sign has been documented in ruptured plaques previously.^22^ We found that although the Napkin-Ring sign occurred more frequently in ruptured than non-ruptured plaque, it was also present in 31% of intact plaques. Whilst the Napkin-Ring sign in ACS may be due to thrombus (e.g. after plaque rupture) surrounded by contrast agent, it may also occur in intact plaques due to large necrotic cores.

Unlike previous studies, coronary CTA were performed within 24 h of invasive angiography and IVUS so there was no time delay for healing of disrupted plaque. It has been suggested that small calcific elements could be mistaken for intra-plaque contrast, especially in the absence of a pre-contrast scan.^16^ In our study, all three false-positive rupture identifications occurred in plaques with spotty calcification emphasising that it can be difficult to distinguish between small calcific plaques and contrast pools (Fig. 1).

This study also sought to determine the ability of coronary CTA features to identify potentially vulnerable VH-IVUS defined plaque types. Plaques found to contain a confluent necrotic core by VH-IVUS are termed fibroatheroma and their presence were associated with adverse advents in prospective studies.^17^, ^14^ The presence of LAP on coronary CTA identified VH-IVUS-defined fibroatheromas with a sensitivity of 76% and specificity of 64%, well below that required for clinical utility. Interestingly using the Napkin-Ring sign increased specificity to 100% but reduced sensitivity to 56%. This is likely due to the spatial resolution of coronary CTA being lower than VH-IVUS. Previously coronary CTA has a shown a sensitivity of 75% to detect necrotic core of >2 mm^2^ and 55% if < 2 mm^2^ when validated against post-mortem histology.^6^ However, autopsy data shows that average necrotic core sizes of ruptured plaques are 2.2 mm^2^,^18^ indicating that detection of smaller necrotic cores may be less clinically relevant.

Coronary CTA identification of VH-IVUS-defined thin-cap fibroatheromas (TCFA), the plaque type at the greatest risk of causing future events^19^, ^14^, ^20^ would improve risk stratification. None of the conventional coronary CTA high-risk features (RI, LAP, Napkin-Ring sign, spotty calcification and non-calcified plaque volume) were different between VH-IVUS-defined thin and thick-cap fibroatheromas. Autopsy studies have shown that TCFA tend to have a higher ratio of necrotic core compared with fibrous plaque.^4^, ^18^ However, attempts to identify necrotic core on the basis of a fixed x-ray attenuation cut-off have previously been limited by the change in the attenuation of plaque components with different contrast intensities.^21^ In this study we measured the volumes of plaque components by creating Plaque Maps based on the ratio of contrast to plaque attenuation using cut-offs previously calculated using post-mortem histology that adjust for inter-patient variation in contrast intensity.^6^ Using this, we found the ratio of necrotic core to fibrous plaque (NC/Fib ratio) was significantly increased in thin-cap (0.90 ± 0.40) vs. thick-cap fibroatheromas (0.59 ± 0.27), p < 0.01. Using ROC curve analysis, the AUC for NC/Fib was 0.77, giving a sensitivity and specificity to detect TCFAs of 84% and 75% at a NC/Fib ratio cut off of >0.58, and 33% and 92% at >1.01.

Finally, this study compared coronary CTA of plaques responsible for ACS vs. stable angina. Consistent with previous studies,^7^, ^23^, ^13^, ^9^ ACS lesions had increased spotty calcification, LAP, Napkin-Ring sign, positive remodeling index, and non-calcified plaque volume. Using Plaque Map-determined quantification of plaque components expanded the number and type of higher-risk criteria on coronary CTA. In particular, ACS culprit plaques contained a higher percentage of necrotic core and larger NC/Fib ratios.

## 5 Limitations

This study has some limitations. First, as it is an *in-*vivo study, plaques were defined by VH-IVUS rather than the gold standard of histology; however whilst VH-IVUS lacks the resolution to directly measure cap thickness and hence should not be considered directly analogous with ex-vivo histology, the VH-IVUS definitions we used for both thin and thick-cap fibroatheromas are widely accepted and have been validated *ex vivo* against histology^5^, ^6^ and identify high-risk lesions in prospective studies.^19^, ^14^, ^20^ Secondly, when examining plaque that has caused ACS with coronary CTA it is possible that any thrombus present may be confused with low attenuation plaque and miss-represented as necrotic core. Finally, when defining plaque rupture using Plaque Maps, small areas of calcified plaque may have been mistaken for intra-plaque contrast. If non-contrast scans were acquired for comparison the specificity to detect plaque rupture might have been higher. In addition, it is possible that pools of intra-plaque contrast may actually represent old ulceration, rather than acute plaque rupture.

## 6 Conclusions

We investigated the ability of coronary CTA features to identify high-risk plaque. We found that when compared with intravascular ultrasound, intra plaque contrast on coronary CTA identifies ruptured plaque with high specificity but low sensitivity and the “Napkin-Ring” sign was very specific at identifying necrotic core but only moderately sensitive. We also show that CT ‘Plaque Map’ quantification of plaque composition (in particular necrotic core/fibrous plaque ratio) can differentiate between levels of VH-IVUS-defined plaque vulnerability (thin-cap vs. thick-cap fibroatheroma) *in vivo* where traditional markers of CT plaque vulnerability could not. Plaque Maps derived necrotic core/fibrous plaque ratio is a new index of vulnerability that needs evaluation in prospective studies.

## Conflict of interest

None.
